# Thymol and Carvacrol Downregulate the Expression of *Salmonella typhimurium* Virulence Genes during an In Vitro Infection on Caco-2 Cells

**DOI:** 10.3390/microorganisms8060862

**Published:** 2020-06-07

**Authors:** Giulia Giovagnoni, Barbara Rossi, Benedetta Tugnoli, Federico Ghiselli, Andrea Bonetti, Andrea Piva, Ester Grilli

**Affiliations:** 1 DIMEVET, Dipartimento di Scienze Mediche Veterinarie, Università di Bologna, Via Tolara di Sopra 50, 40064 Ozzano dell’Emilia (BO), Italy; giulia.giovagnoni4@unibo.it (G.G.); federico.ghiselli4@unibo.it (F.G.); andrea.bonetti15@unibo.it (A.B.); andrea.piva@unibo.it (A.P.); 2 Vetagro S.p.A., via Porro 2, 42124 Reggio Emilia, Italy; barbara.rossi@vetagro.com (B.R.); benedetta.tugnoli@vetagro.com (B.T.); 3 Vetagro Inc., 116 W. Jackson Blvd., Suite #320, Chicago, IL 60604, USA

**Keywords:** *Salmonella typhimurium*, tight junctions, epithelial barrier, virulence genes, nature identical compounds, thymol, carvacrol, antimicrobial activity, feed additives

## Abstract

*Salmonella typhimurium* is one of the major bacteria responsible for gastroenteritis in humans caused by foodborne pathogens. As pork is one of the main routes of transmission, bioactive compounds used as feed additives may be an important strategy to control *Salmonella typhimurium*. The aim of this study was to assess the antimicrobial activity of several organic acids and nature identical compounds against *Salmonella typhimurium* ATCC^®®^ 6994™. Moreover, the effect of sub-lethal concentrations of thymol and carvacrol in counteracting a *Salmonella typhimurium* in vitro infection on Caco-2 cells was evaluated, focusing on the maintenance of the epithelial barrier and the alteration of *Salmonella* virulence genes. The results showed a protective effect of the compounds on the integrity of the intestinal monolayer, improving transepithelial electrical resistance and bacterial translocation compared to the non-treated cells. A real-time PCR study highlighted a significant downregulation of the main virulence genes of *Salmonella* (*hilA*, *prgH*, *invA*, *sipA*, *sipC*, *sipD*, *sopB*, *sopE2*). These findings indicate that thymol and carvacrol could be good candidates for the control of *Salmonella typhimurium* in pigs.

## 1. Introduction

*Salmonella typhimurium* is a Gram-negative foodborne pathogen, reported as the second and third most common cause of human gastrointestinal infections in Europe and the United States, respectively [1,2]. Among European cases, 27.3% of the isolated strains were associated with matrices of porcine origin, confirming this species as one of the main pathways of transmission to humans [1,3]. *S. typhimurium* infection in pigs must not be underestimated, since it can cause enterocolitis, but it can also persist asymptomatically, making the pig an important reservoir of the pathogen, reducing productivity and average daily gain [4,5]. In the latter case, *S. typhimurium* colonizes the gastrointestinal tract of the pig in a persistent and chronic way and, during periods of stress, this asymptomatic colonization often flares up [6]. The most dangerous situations are during transport and at the slaughterhouse, where the spread of this pathogen is amplified and the contamination of food through fecal matter becomes the main route of transmission to humans [7,8].

The pathogenesis of *Salmonella* spp. is widely studied and it is known to be associated with *Salmonella* pathogenicity islands (SPI) [9]. SPI-1 and SPI-2, in particular, encode for two type III secretion systems (T3SS), a complex apparatus composed of structural, translocator and effector proteins that allow the entry and the maintenance of bacteria in the host intestinal cell [10]. Structural proteins are responsible for the assembly of the T3SS apparatus, formed by two rings crossing the inner and outer bacterial membrane, linked to a needle-like system that pierces the host cellular membrane [10,11]. The latter structure is crucial for the transport of the effector proteins, delivered by translocators from the bacterial cell into the host: here, effectors manage fundamental changes enabling key steps for *Salmonella* pathogenesis [12]. During the bacterial infection, *Salmonella* invade the enterocyte and survive into the host cytoplasm surrounded by large vesicles, called *Salmonella*-containing vacuoles (SCV) [13]. The disruption of the intestinal epithelial barrier, as a consequence of the disruption of tight junctions (TJ), is the main issue of the infection, giving rise to diarrhea-generating gastroenteritis. The Caco-2 cell monolayer system is a proven in vitro model for studying the structure and functionality of TJ, even during bacterial challenges [14,15]. In fact, most enteric pathogens are known to perturb the intestinal epithelial barrier, impacting transepithelial electrical resistance (TER) or paracellular permeability, and altering the arrangement of TJ with different mechanisms. It has been demonstrated that several effector proteins encoded by SPI-1, such as SipA, SopB, SopE, and SopE2, are responsible for alteration of these parameters [16].

Nowadays, it is crucial to use alternatives to antibiotics in the control of *S. typhimurium*. Nature identical compounds (NIC) have been widely studied for their antibacterial properties [8,17], which makes them suitable candidates as feed additives.

The aim of the study was to investigate the role of sub-lethal concentrations of thymol and carvacrol in counteracting a *S. typhimurium* infection of Caco-2 cells, focusing on the maintenance of the epithelial barrier and the alteration of *Salmonella* virulence genes.

## 2. Materials and Methods

### 2.1. Bacterial Strain and Culture Conditions

The strain used in this study was the ATCC^®®^ *Salmonella typhimurium* 6994™. *S. typhimurium* was conserved at −80 °C in Brain Heart Infusion broth (BHI) supplemented with 20% (*v*/*v*) glycerol, and it was recovered in BHI at 37 °C. The identification was carried out using Microbact™ 24E (Oxoid, Basingstoke, United Kingdom).

### 2.2. Chemicals and Test Solutions

Citric acid, sorbic acid, benzoic acid, butyric acid, hexanoic acid, thymol, vanillin, carvacrol, and eugenol were purchased from Alfa Aesar (Thermo Fisher GmbH, Kandel, Germany). Stock solutions of organic acids and NIC were prepared in BHI and BHI supplemented with 70% (*v*/*v*) ethanol, respectively. All solutions were buffered to pH 6.5 and filter-sterilized, then they were conserved at +4 °C and brought back to room temperature before each use.

### 2.3. Minimal Inhibitory Concentration of Bioactive Compounds

The minimal inhibitory concentrations (MIC) of organic acids and NIC were determined in triplicate using the broth microdilution method in 96-well microtiter plates. Citric acid, sorbic acid, benzoic acid, butyric acid, and hexanoic acid were tested at final concentrations ranging from 100 to 1.56 mM (2-fold dilutions), whereas thymol, vanillin, carvacrol, and eugenol from 7.5 to 0.12 mM (2-fold dilutions). An overnight bacterial culture of *S. typhimurium* was diluted to obtain an inoculum of 10^6^ CFU/mL. For each bioactive compound, the MIC value was defined as the lowest concentration that resulted in null absorbance (630 nm) registered with Varioskan™ LUX Multimode Microplate Reader (Thermo fisher Scientific Inc., Waltham, MA, USA) after 24 h of incubation at 37 °C.

### 2.4. Time-Kill Curve of Thymol and Carvacrol

A time-kill assay was performed in order to evaluate the bactericidal effect of thymol and carvacrol against *S. typhimurium* over time. *S. typhimurium*, diluted at 10^6^ CFU/mL in BHI pH 6.5 from an overnight culture, was incubated at 37 °C for 24 h without any substance (CTR), and either with thymol or carvacrol at 1.87 (MIC), 0.94, and 0.47 mM. Bacteria were counted at the inoculation time (0 h), every hour for the following 8 h, and then after 24 h of incubation. At each time-point, a 100 µl aliquot was collected from each tube and the samples were serially (10-fold) diluted in saline solution. Dilutions were then plated on BHI agar plates and incubated at 37 °C for 24 h.

### 2.5. Effect of Thymol and Carvacrol on Intestinal Caco-2 Cells Monolayer Integrity

The human colon adenocarcinoma cell line (Caco-2) was obtained from DSMZ (DSMZ-German Collection of Microorganisms and Cell Cultures, Leibniz Institute, Braunschweig, Germany). Caco-2 cells were seeded at a density of 1.5 × 10^5^ cells/well onto 12 well transwell polyethylene terephthalate inserts (3.0 µm pore; Corning Incorporated, Corning, NY, USA) and were maintained at 37 °C, in an atmosphere containing 5% CO_2_ at 95% relative humidity, in DMEM supplemented with 10% fetal bovine serum (FBS), 1% non-essential amino acids (NEAA), 1% penicillin/streptomycin (P/S) and 1% L-Glutamine. The medium was replaced every other day, both before and after cells reached confluence. Moreover, confluent cells were monitored measuring transepithelial electrical resistance (TER) at the same time as the change of medium, using an epithelial tissue voltohmmeter (Millicell ERS-2, Merck KGaA, Darmstadt, Germany).

Cells were used for bacterial challenge 28 days after the seeding on filters, once TER values were stable. On day 28, TER was measured right before the start of the challenge and the cellular medium was removed and substituted with the same medium without P/S. Cells were apically challenged with *S. typhimurium* in exponential phase at a concentration of 10^5^ CFU/mL with EtOH in a concentration equivalent to that found in the treated groups (challenged control, CTR+), and with either thymol or carvacrol at 0.47 mM (*n* = 5). A group in the same conditions of CTR+, but without bacterial challenge, was identified as the non-challenged control (CTR−). In order to determine the intestinal monolayer integrity and the infection process, TER and bacterial translocation were examined during a 4 h challenge at 2 and 4 h. For bacterial translocation, a 100 µL aliquot of the basolateral medium was collected, serially (10-fold) diluted in saline solution and seeded on BHI agar plates in order to count how many bacteria translocated from the apical to the basolateral side of the intestinal monolayer.

### 2.6. Determination of Salmonella Virulence mRNA Expression

mRNA expression analyses were performed by extracting RNA from *S. typhimurium* cultures with thymol and carvacrol. In brief, 10^6^ CFU/mL of *S. typhimurium* was incubated for 4 h at 37 °C with thymol or carvacrol at 0.47 mM. A control group with and without ethanol was also performed (EtOH; CTR). After incubation, the bacterial cultures were centrifuged for 5 min at 5000× g and the supernatants were discarded. The pellets were resuspended in 100 µL of TE buffer supplemented with 1 mg/mL of lysozyme and incubated for 10 min at 37 °C. NucleoSpin^®®^ RNA Kit (Macherey-Nagel GmbH & Co. KG, Düren, Germany) was used for RNA extraction and purification in accordance with manufacturer’s instructions. RNA yield and quality were verified spectrophotometrically using A230, A260, and A280 nm measurements (μDrop™ Plate and Varioskan™ LUX, Thermo fisher Scientific Inc., Waltham, MA, USA). The extracted RNA was converted into cDNA using iScript™ cDNA Synthesis Kit (Bio-Rad Laboratories, Inc., Hercules, CA, USA) according to manufacturer’s instructions.

After cDNA synthesis, *S. typhimurium* virulence gene expression was determined with a real-time PCR, testing the genes listed in Table 1, using CFX96™ Real-Time PCR Detection System (Bio-Rad Laboratories, Inc., Hercules, CA, USA). The reaction contained 5 μL of 2× iTaq™ Universal SYBR^®®^ Green Supermix (Bio-Rad Laboratories, Inc., Hercules, CA, USA), 200 or 600 nM of each primer, 2 μL of 5 ng/μL cDNA, and nuclease-free water up to the final volume of 10 μL. The samples were analyzed under the following conditions: 3 min at 95 °C, followed by 40 cycles of 95 °C for 10 s and 60 °C for 30 s. Specificity of each reaction was evaluated by melting-curve analysis with 0.5 °C/s heating rate from 55 up to 95 °C.

mRNA expression was normalized using *rpoD* as housekeeping gene. After determining the threshold cycle (Ct) for each gene, the relative changes in mRNA expression of *S. typhimurium* grown with thymol and carvacrol compared to controls were calculated using the 2^−ΔΔCt^ method [18].

### 2.7. Statistical Analysis

The data were analyzed with GraphPad Prism v. 8.4.1 (GraphPad Software, Inc., San Diego, CA, USA) and differences were considered significant at *p* ≤ 0.05. For cell culture and mRNA expression data, two-way ANOVA was performed, followed by the Tukey post hoc test for multiple comparisons.

## 3. Results

### 3.1. Thymol and Carvacrol Have the Lowest MIC among Tested Bioactive Compounds

**Figure 1:**
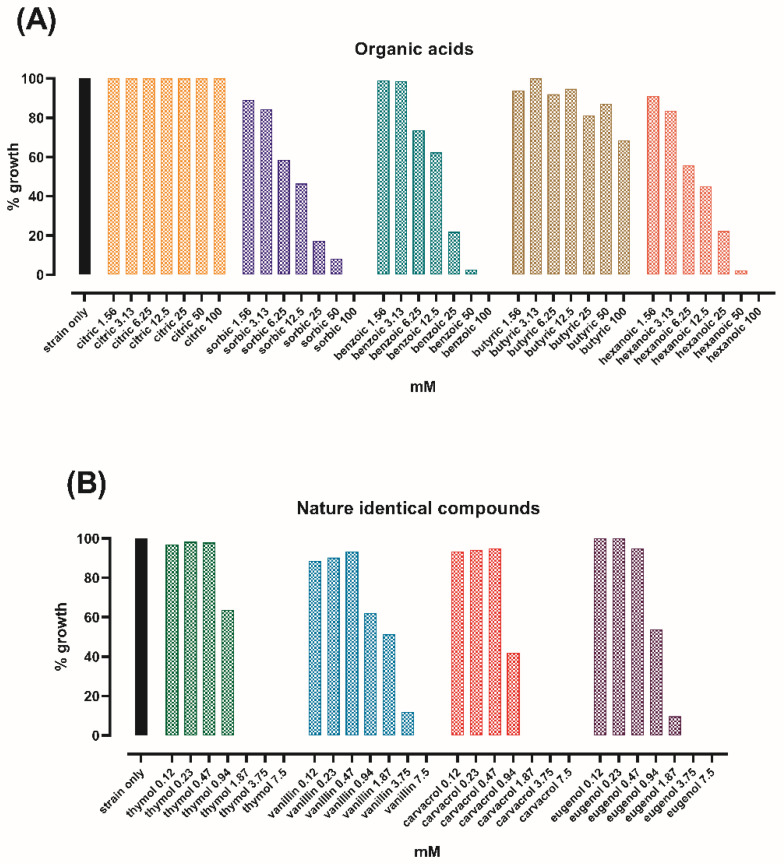
*Salmonella typhimurium* growth after 24 h in the presence of organic acids (**A**) or nature identical compounds (**B**). Bacterial growth is expressed as a percentage relative to the control (strain only).

The antimicrobial activity of organic acids and NIC against *S. typhimurium* are reported in Figure 1A,B, respectively. Citric and butyric acid did not inhibit *S. typhimurium*, while the MIC value of sorbic, benzoic, and hexanoic acid was defined as 100 mM. All the tested NIC were effective at lower concentrations than organic acids. In particular, vanillin inhibited *S. typhimurium* at 7.5 mM, eugenol at 3.75 mM, and both thymol and carvacrol at 1.87 mM.

### 3.2. The Bactericidal Effect of Thymol and Carvacrol Is Dose-Dependent

**Figure 2:**
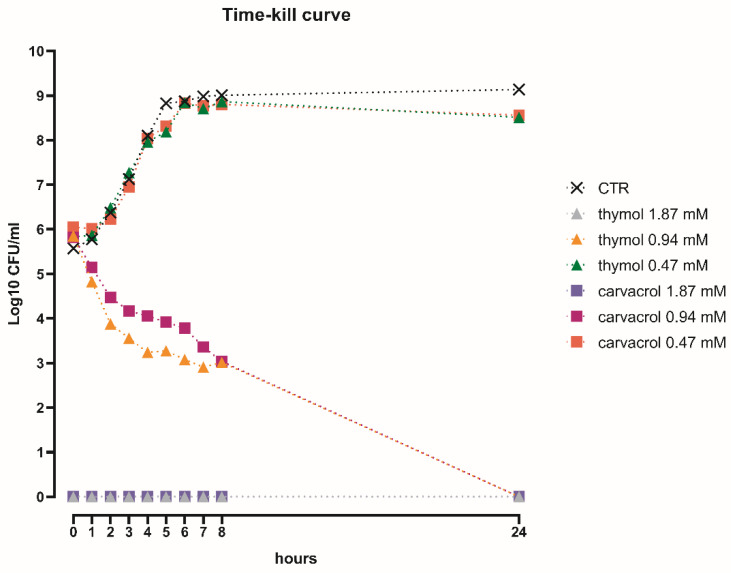
Time-kill curve of thymol or carvacrol 1.87, 0.94, and 0.47 mM against *S. typhimurium*.

The time-kill study highlighted a dose-dependent effect over time for both thymol and carvacrol, as shown in Figure 2. The highest concentrations tested (1.87 mM), corresponding to the MIC value defined by the microdilution method, had a strong bactericidal effect against *S. typhimurium* immediately after inoculation. Thymol and carvacrol at 0.94 mM gradually reduced Salmonella viability during the first 8 h, to reach a complete kill within 24 h. Finally, the two NIC at 0.47 mM did not have any direct antibacterial activity, since the growth of *S. typhimurium* was comparable to the control without substances.

### 3.3. Thymol and Carvacrol at Sub-Lethal Concentrations Can Counteract a S. typhimurium Challenge on Caco-2 Cells

**Figure 3:**
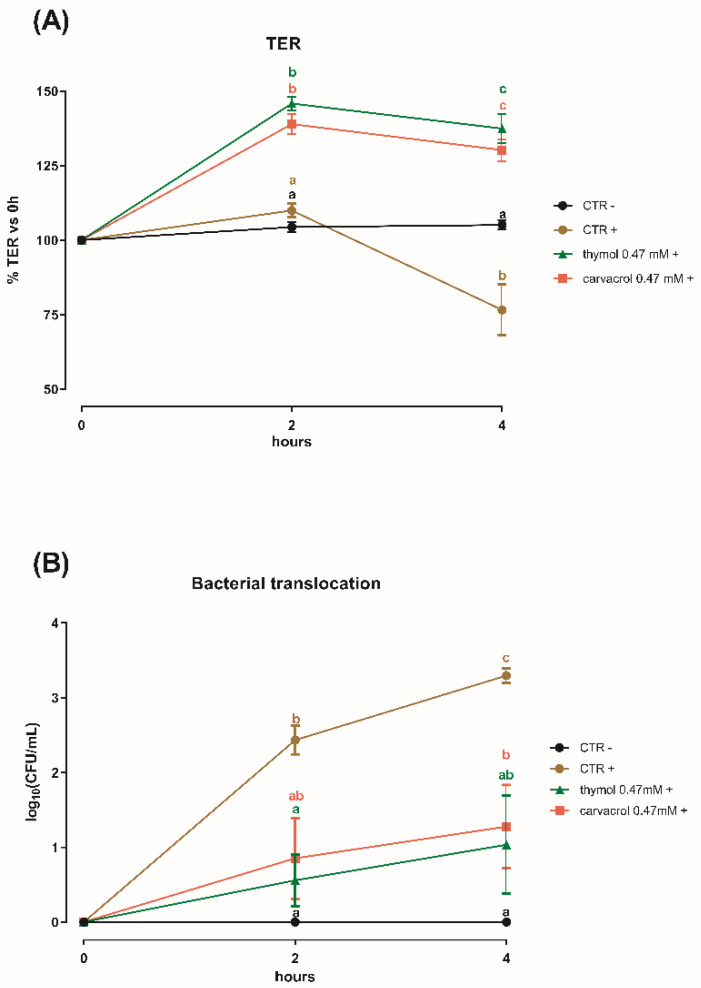
(**A**) Transepithelial electrical resistance (TER) and (**B**) bacterial translocation of Caco-2 cells cultured with ethanol (CTR−; CTR+), thymol, or carvacrol 0.47 mM post-challenge with *S. typhimurium* (+), or without bacterial challenge (−). Data are presented as mean ± SEM. Different letters indicate statistical significance with *p* < 0.05.

Results of transepithelial electrical resistance (TER) and bacterial translocation are represented in Figure 3A,B, respectively. The challenge with *S. typhimurium* on Caco-2 cells induced a drop in TER and an increase in bacterial translocation. After 2 h no differences were shown in the measurement of TER between the two control groups, while thymol and carvacrol increased TER by more than 30% (*p* < 0.0001). At 4 h post-challenge, CTR+ showed a 25% drop in TER. Cells treated with thymol or carvacrol maintained a higher TER compared to both CTR+ and CTR− (*p* < 0.0001).

Differences were also observed for bacterial translocation. After 2 h, CTR+ results were significantly different compared to CTR− and the thymol treated groups (*p* < 0.05). At 4 h, bacteria counted in the basolateral side of both NIC treated cells were significantly lower than in the CTR+ (*p* < 0.05); however, thymol at 0.47 mM was comparable to the non-challenged control at both time-points.

### 3.4. Sub-Lethal Concentrations of Thymol and Carvacrol Downregulate Virulence Genes of S. typhimurium

**Figure 4:**
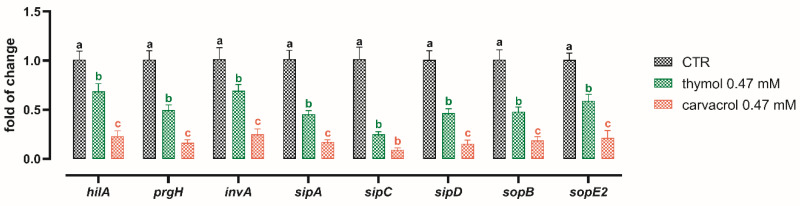
mRNA expression of *hilA*, *prgH*, *invA*, *sipA*, *sipC*, *sipD*, *sopB*, *sopE2* from *S. typhimurium* cultures alone (CTR), and with either thymol or carvacrol 0.47 mM. Data are presented as mean ± SEM. Different letters indicate statistical significance with *p* < 0.05.

All of the *S. typhimurium* virulence genes that were investigated were downregulated by thymol and carvacrol (Figure 4). No differences were found between the two control groups (CTR and EtOH) and for this reason EtOH data are not shown. Thymol and carvacrol decreased the expression of all the genes, compared to CTR (*p* < 0.05). Moreover, mRNA expression was significantly lower in treatment with carvacrol than thymol, except for *sipC*.

## 4. Discussion

*S. typhimurium* is one of the major foodborne pathogens worldwide. Since pigs are usually asymptomatic, the main concern related to swine salmonellosis is the sly transmission to humans through pork products. In order to prevent contamination of animal products at the slaughterhouse, the key is to prevent the colonization of pigs during the production cycle. In the past years, several strategies have been studied for the reduction of *Salmonella* prevalence in swine farms, including measures of hygiene and biosecurity, vaccine programs, and feeding practices [22]. Focusing on feeding, the main principle is the balancing of intestinal microflora in favor of beneficial bacteria, avoiding the harmful colonization of *Salmonella*. For this purpose, in the last decades several feed additives were adopted, as organic acids and botanicals, thanks mainly to their antimicrobial properties [23,24]. In fact, the role of organic acids in killing harmful bacteria, as well as in the alteration of *Salmonella* gene expression, has been investigated for many years [25]. Although herbal extracts are a relatively new approach compared to acids, the antimicrobial action of these compounds is equally powerful [26].

The approach of this study was to perform a preliminary screening of organic acids and NIC with the aim to detect the molecules with the greatest direct antimicrobial activity against *S. typhimurium*. This first screening revealed that NIC had stronger antimicrobial activity than organic acids, which is consistent with other reports [27,28,29], and this would offer an explanation in the theory of acid tolerance of *S. typhimurium* in response to low pH [30]. Then, as thymol and carvacrol were identified as the most effective molecules, they were used in a time-kill assay, with the purpose of assessing sub-inhibitory concentrations to test on cell cultures. The protective effects of thymol and carvacrol on the in vitro intestinal monolayer were verified by measuring functionality and integrity parameters (TER, bacterial translocation) of Caco-2 cells infected with *S. typhimurium*.

Caco-2 cells, together with other human adenocarcinoma cell lines, mimic the intestinal epithelium by polarizing on permeable filters [31]. This system is suitable for bacterial challenges that interfere with the intestinal monolayer, and it is a standardized approach for studying *S. typhimurium* invasivity [15]. In the model of Finlay and Falkow [15], *S. typhimurium* (10^7^) was added on 10–14 day-old Caco-2 cells on 3 um filters. Authors were able to observe bacteria invading host cytoplasm enclosed in SCV by scanning and transmission electron microscopy. Moreover, consistent with our results, bacteria appeared in the basolateral medium 2 h post-challenge, while transepithelial electrical resistance (TER) dropped after 3–4 h, suggesting that our challenge worked correctly. In this study, thymol and carvacrol were able to counteract the bacterial challenge by increasing TER values even beyond the non-challenged control and maintaining the high values throughout the experiment. Transepithelial electrical resistance is an indicator of the health status and the tightness of the intestinal epithelium [32], and it is reported that many NIC have anti-inflammatory and antioxidant activity, that in turn can improve gut morphology and integrity as mediated by tight junction proteins [33]. In parallel, thymol and carvacrol also reduced bacterial translocation across the monolayer. Several studies investigated the role of carvacrol against the invasion of IPEC-J2 cells by *S. typhimurium* [34,35]. Inamuco et al. [34] found that carvacrol 0.5 mM significantly reduced the invasion of porcine epithelial cells, probably due to the loss of functionality of flagella. A subsequent study by Burt et al. [35] confirmed the results obtained by Inamuco et al., assuming that virulence factors could be affected by carvacrol. In a study conducted by Zhang et al. [36], thymol 0.2 mM significantly inhibited the translocation of SipA in an in vitro infection of HeLa cells. This finding indicates that the inhibition of T3SS is the primary protective effect of sub-lethal concentrations of thymol. These data are confirmed in our study, as the same concentration of thymol reduced cell invasion by reducing virulence gene expression, including SipA. Bacteria have, among their survival strategies, the flexibility of altering the mRNA expression patterns in response to environmental stress [37], as for example when cultured with non-lethal concentrations of antimicrobials. Since the mechanism of action of NIC involves the alteration of the bacterial cell membrane and induces an intracellular ATP leakage [29,38], it can be postulated that bacteria subjected to stressful sub-lethal concentrations of NIC switch their energies from motility or virulence systems to vital and essential processes, until a more favorable environment is restored [39]. Recently, it has been demonstrated that a stress induced by thymol influences the *S. typhimurium* proteome, downregulating genes involved in chemotaxis, motility, and virulence [20]. In addition, it is reported that these substances can affect quorum sensing of bacteria [40], which, in turn, controls virulence factor production [41,42].

It is plausible that the amelioration of TER and the reduction of bacterial translocation across Caco-2 cells observed in this study was mediated by the downregulation of a panel of several virulence genes that are involved in a complex series of reactions necessary to exploit *Salmonella* pathogenicity. In particular, we observed a reduction of the expression of the transcriptional activator of invasion *HilA*, which is the first trigger and activates genes responsible for the assembly of the T3SS structure, including *prgH* and *invA* [11]. *HilA* upregulation was correlated with tetracycline resistance cases [19], whereas its downregulation is attributed to several bioactive compounds such as short and medium chain fatty acids [43,44]. This would support our findings suggesting a possible cascade effect on the expression of *prgH* and *invA*. The other genes that were significantly downregulated by thymol and carvacrol are all initiators of a cascade of reactions that ultimately ends in cytoskeletal rearrangements and the disruption of TJ proteins, with a consequent increase in paracellular permeability [16,45,46]. Ultimately, this would facilitate the passage of *Salmonella* from the apical side to the basolateral side, thereby ending the *Salmonella* vicious cycle with a worsening of its pathogenicity [47].

## 5. Conclusions

The data from this study support a dual mechanism of action of thymol and carvacrol in ameliorating the effects associated with a *S. typhimurium* in vitro challenge. From the host side, thymol and carvacrol have anti-inflammatory and anti-oxidant properties that can prevent the cascade of inflammatory cytokines due to *Salmonella* infection, and contribute to the maintenance of the epithelial integrity, whereas from the pathogen side these molecules have direct antimicrobial properties that can inhibit the growth of *Salmonella*, but at the same time at sub-inhibitory concentrations they can alter a set of virulence genes that are responsible for invasion and damage to the epithelium. Since thymol and carvacrol are already widely used as feed additives, after confirming these findings also in vivo, these molecules could be helpful candidates in the control of salmonellosis in pigs.

## Figures and Tables

**Table 1:** List of primers used for real-time PCR.

Gene	Function	Sequence (5′ → 3′)	Product Length (bp)	Accession Number	Reference
*hilA*	Transcriptional regulator of the SPI-1 gene expression	F: CTGTACGGACAGGGCTATCG R: GCAGACTCTCGGATTGAACC	130	U25352.1	[19]
*prgH*	Assembly of base ring structure of T3SS	F: CGCTGCGCAAAATGAAAGAG R: TTACGCGGCTCATCGAAATG	177	U21676	[20]
*invA*	Assembly of needle-like structure of T3SS	F: TCTGGATGGTATGCCCGGTA R: TCATCGCACCGTCAAAGGAA	140	M90846.1	This study
*sipA*	Actin rearrangement	F: GTCATTCGCGTGTGGATTCG R: TTCGGATGAAGCGTTGGTCA	143	U40013.1	This study
*sipC*	Actin rearrangement	F: ACGGGCAGAATAGCGTCAAA R: ATACCCAGACTTTCCGTGGC	150	U25631.1	This study
*sipD*	Translocation of effector proteins	F: ATTCCGCTTCTCCTCATCCG R: ACCGCGATGTTCTGTGGTAG	107	U40013.1	This study
*sopB*	Invasion, generation, and maintenance of SCV	F: GATGGCGGCGAACCCTATAA R: GAAGACTACCAGGCGCACTT	181	AF213335.2	This study
*sopE2*	Actin rearrangement	F: GAACGCTTCTGAGGGTAGGG R: CGAGCATAGGCCGGATCTTT	117	AF200952.1	This study
*rpoD*	Housekeeping	F: GTGAAATGGGCACTGTTGAACTG R: TTCCAGCAGATAGGTAATGGCTTC	131	NC_003197.2	[21]
